# Hughes-Stovin Syndrome: a case report and review of the literature

**DOI:** 10.1186/1757-1626-2-98

**Published:** 2009-01-29

**Authors:** Athanasios N Chalazonitis, Stefanos B Lachanis, Panagiotis Mitseas, Panagiotis Argyriou, Joannie Tzovara, Petros Porfyrides, Evangelia Sotiropoulou, Nikolaos Ptohis

**Affiliations:** 1 Alexandra' University Hospital, Department of Radiology, Vas Sofias ave 80, 11528, Athens, Greece; 2 401 Army General Hospital, Department of Radiology, Mesogeion ave 138, 115 25 Athens, Greece

## Abstract

**Background:**

Hughes-Stovin syndrome is a rare entity. The aetiology of Hughes-Stovin syndrome is still unknown and the natural course of the illness is usually fatal; however it is supposed to be a clinical variant manifestation of Behçet disease.

**Case presentation:**

We report the case of an 18 years old, greek male patient with Hughes-Stovin syndrome, who initially presented with deep vein thrombosis. There were no findings consistent with Behçet disease and the haemoptysis was treated successfully with methylprednisolone. Pathogenesis, imaging investigation and treatment of this syndrome are also briefly discussed.

**Conclusion:**

In young men presenting with venous thrombosis as revealed on imaging examination, with platelet count and coagulation tests within normal and hemoptysis the eventuality of Hughes-Stovin syndrome is to be considered.

## Background

Hughes-Stovin syndrome is a rare condition first described in 1959 [1] which is characterized by multiple pulmonary artery aneurysms and peripheral venous thrombosis. Typical symptoms are recurrent fever, chills, haemoptysis and coughs and it usually affects young men. The natural course of the illness is usually fatal because of fulminant haemoptysis. The aetiology of Hughes-Stovin syndrome is still unknown; however it is supposed to be a clinical variant manifestation of Behçet disease.

## Case presentation

**Figure 1:**
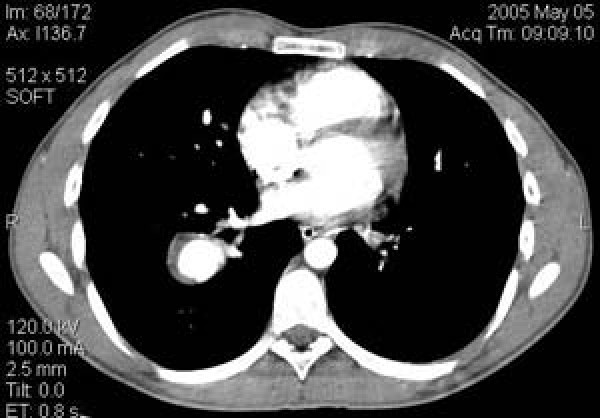
**Chest Contrast Enhanced-MSCT angiography axial section showing a partially thrombosed aneurysm of the right lower lobe pulmonary artery, and thrombosed segmental branch of the left lower lobe pulmonary artery**.

**Figure 2:**
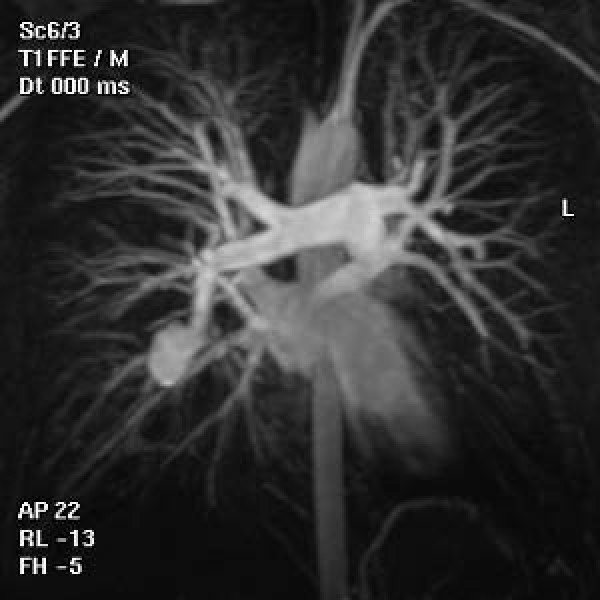
**Contrast Enhanced pulmonary MRA (coronal MIP) showing aneurysm of the right lower lobe pulmonary artery**.

**Figure 3:**
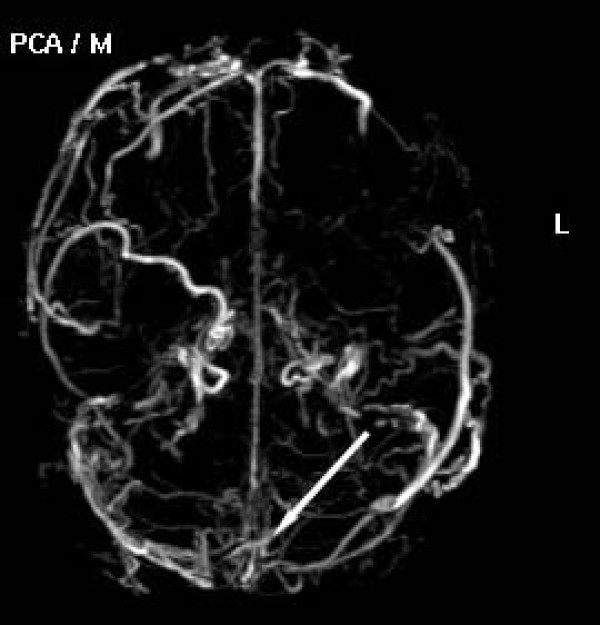
**Axial MIP of 3D-PC MRV showing chronic thrombosis of superior sagittal and transverse sinuses (arrows)**.

An 18 years old, Greek male patient presented with edema of his left leg. His vital signs including temperature, arterial pressure and heart rate were normal. Clinical examination was negative. A color Doppler examénation showed deep vein thrombosis and subsequently heparin therapy begun. Contrast Enchanced Computed Tomography (CT) of the abdomen and pelvis was also performed in order to exclude a major intra-abdominal malignancy and thrombosis of left external iliac vein was depicted as well. A history of migraine during the last 3 years and anorexia nervosa was also reported. After 10 days on heparin therapy, dry cough begun, followed by an episode of moderate haemoptysis (100–120 ml). At review his vital signs were normal and his platelet count and coagulation tests were within normal limits. There was no leukocytosis, and the erythrocyte sedimentation rate was normal. Skin examination was normal. Chest postero-anterior plain film showed a nodular opacity in the mid right lung field. Chest Contrast Enchanced-Multislice CT (MSCT) and Magnetic Resonance angiography (MRA) revealed a partially thrombosed aneurysm of the right lower lobe pulmonary artery and left lower lobe pulmonary artery occlusion (Fig. 1, 2). Lung window settings of the MSCT revealed a right lower lobe ground glass opacity probably representing a pulmonary hemorrhage, while left lower lobe parenchyma appeared normal. MR brain venography showed chronic thrombosis of superior sagittal and transverse sinuses (Fig. 3).

Finally, a diagnosis of Hughes-Stovin syndrome, was made on the basis of pulmonary artery aneurysms and venous thromboses in such a young patient, without clinical findings consistent with Behçet disease. Even though our patient lacked a history of fever and chills, however dry cough, followed by haemoptysis due to pulmonary aneurysm and peripheral venous thrombosis without any clinical manifestations of Behçet disease were strong evidence of Hughes-Stovin diagnosis. Haemoptysis resolved with intravenous methylprednisolone treatment after about 25 days.

## Discussion

Hughes-Stovin syndrome is a rare clinical condition characterized by multiple pulmonary artery aneurysms and peripheral venous thrombosis, first described in 1959 [1]. Patients, mostly men aged 12–40 years may present with haemoptysis, cough, dyspnea, chest pain, and signs of pulmonary hypertension. Other associated features reported in Hughes-Stovin syndrome include fever and elevated intracranial hypertension Since its first description, there have been only a few reports of its occurrence in the English medical literature [2]. Aneurysms usually involve the pulmonary arteries and the bronchial arteries resulting in subsequent haemoptysis. However they can occur anywhere in systematic circulation [3]. Recurrent phlebitis also commonly involves the large vessels resulting in thrombus formation. In general there is a thrombus formation predisposition affecting the peripheral veins. Thrombosis of the vena cava and of the right atrium has also been described [2,4]. Histologic studies show destruction of the arterial wall and perivascular lymphomonocytic infiltration of capillaries and venules [5].

The pathogenesis of Hughes-Stovin syndrome is not known. The current consensus opinion is that Hughes-Stovin syndrome results from a vasculitis similar to that implicated in Behçet disease [2]. Vasculitis in Behçet disease results in arterial occlusion, arterial aneurysm, venous occlusion, and varices [6], indicating systemic vessel involvement. Furthermore, it is very likely that Hughes-Stovin syndrome represents a cardiovascular manifestation of Behçet disease, since in both clinical entities pulmonary aneurysms are characteristic clinical features [7]. However other clinical presentations can overlap significantly; both are more common in young men, the pulmonary manifestations of both can be identical, and the histology of the aneurysms in both entities can be similar [8].

Regardless the presence of deep venous thrombosis, the pulmonary artery thrombi may originate in situ, secondary to the pulmonary artery wall inflammation. On initial CT images a rim of enhancement at the filling defects may be depicted; this finding is consistent with inflammation – a finding that is not usually seen in acute or chronic pulmonary embolism [9]. Similarly, the pathogenesis of pulmonary artery aneurysms in Hughes-Stovin syndrome has been attributed to weakening of the vessel wall due to inflammation, which corresponds well to the observation by Ketchum et al, that the aneurysms develop at the locations of prior thrombus and abnormal enhancement [9]. Hughes and Stovin's original theory [1] suggested that degenerative bronchial arteries lead to changes in the vasa vasorum of the pulmonary arteries and the development of aneurysms. The volume-rendered CT images according to Ketchum et al show prominent and tortuous bronchial artery branches that apparently supply a web of smaller vessels at the sites of pulmonary artery wall inflammation even before the actual aneurysm formation [9]. Similarly Mahlo et al. [10] and Herb et al. [3] performed digital subtraction angiography of the bronchial arteries and noted distorted and dilated bronchial arteries with convoluted small branches, findings that correspond to Hughes and Stovin's original theory [9].

Haemoptysis remains a leading cause of death in patients with Behçet disease. Similarly, Hughes-Stovin syndrome is often fatal as a result of massive haemoptysis due to pulmonary/bronchial arterial aneurysm rupture. However systemic bronchial artery hypertrophy secondary to ischemia related to the pulmonary artery occlusion could also be the origin of bleeding [3,10]. Finally, haemoptysis could be attributed secondary to both pre-mentioned mechanisms [11].

Conventional angiography can be used for better evaluation of the pulmonary aneurysms and assessment of angiodysplastic bronchial arteries in Hughes-Stovin syndrome.

However, it cannot be performed in all patients with venous thromboses. Contrast-Enhanced MRA and Contrast-Enhanced MDCTA may provide an alternative [12-14] while 3D volume rendering analysis can ideally visualize the presence of an increased number of morphologically abnormal bronchial arteries even before aneurysm formation [9]. In fact it is reported that multi-detector row helical CT angiography provides more precise depiction of bronchial and nonbronchial systemic arteries than does conventional angiography [14].

The syndrome is most often treated by immunosuppressant, either systemic corticosteroids or cytotoxic agents (a combination of cyclophosphamide and glucocorticoids), in patients without or with small amount of hemoptysis in order to stabilize the pulmonary artery aneurysms [2], or even make them disappear [15]. If pulmonary embolism is present a therapeutic dilemma is often posed in using anticoagulants. Anticoagulation may prevent the progression of pulmonary embolism and resolve vein thrombi, but since it increases the risk and severity of haemoptysis, it is contraindicated. Surgical resection of the affected segments of the lung is to be considered in cases of high risk rupture aneurysms limited to one segment or one lung [7]. However, the high morbidity and mortality associated with surgery, and the frequent bilaterality and multifocality of the pulmonary artery aneurysms at the time of diagnosis, makes transcatheter embolization an alternative to surgery in most cases [2]. Identification of aneurysms in the bronchial arteries should be treated by bronchial artery embolization [3]. Even enlargement of bronchial arteries when recognized, should be treated [11].

## Conclusion

The types of vasculitis involved in Hughes-Stovin syndrome and in Behçet disease seems to be very similar. Though most patients with Hughes-Stovin syndrome will present with haemoptysis and advanced disease, dysplastic bronchial arteries and pathologic enhancement of pulmonary thromboemboli seen on CT pulmonary angiograms should alert the radiologist and raise suspicion of pulmonary vasculitis. Earlier diagnosis could be in some cases crucial, allowing the prevention of the development of potentially life-threatening pulmonary artery aneurysms [9].

## Consent

Written informed consent was obtained from the patient for publication of this case report and accompanying images. A copy of the written consent is available for review by the Editor-in-Chief of this journal.

## Competing interests

The authors declare that they have no competing interests.

## Authors' contributions

AN, SL, PM, PA performed the conception and design of this presentation. The also performed the acquisition, analysis and interpretation of data. They revised the manuscript and made final crucial corrections. ES, NP, PP, JT have been also involved in analysis and interpretation of data as well as in the design of the presentation. They performed manuscript writing and revision.

All authors have given final approval of the version to be published.
